# AN-VR-BE. A Randomized Controlled Trial for Reducing Fear of Gaining Weight and Other Eating Disorder Symptoms in Anorexia Nervosa through Virtual Reality-Based Body Exposure

**DOI:** 10.3390/jcm10040682

**Published:** 2021-02-10

**Authors:** Bruno Porras-Garcia, Marta Ferrer-Garcia, Eduardo Serrano-Troncoso, Marta Carulla-Roig, Pau Soto-Usera, Helena Miquel-Nabau, Laura Fernández-Del castillo Olivares, Rosa Marnet-Fiol, Isabel de la Montaña Santos-Carrasco, Bianca Borszewski, Marina Díaz-Marsá, Isabel Sánchez-Díaz, Fernando Fernández-Aranda, José Gutiérrez-Maldonado

**Affiliations:** 1 Department of Clinical Psychology and Psychobiology, University of Barcelona, Passeig de la Vall d’Hebron 171, 08035 Barcelona, Spain; brporras@ub.edu (B.P.-G.); martaferrerg@ub.edu (M.F.-G.); helena.mn29@gmail.com (H.M.-N.); laurafdez_castillo@hotmail.com (L.F.-D.c.O.); marnetfm@gmail.com (R.M.-F.); 2 Department of Child and Adolescent Psychiatry and Psychology, Hospital Sant Joan de Déu of Barcelona, Passeig de Sant Joan de Déu, 2, 08950 Esplugues de Llobregat, Barcelona, Spain; eserrano@sjdhospitalbarcelona.org (E.S.-T.); mcarulla@sjdhospitalbarcelona.org (M.C.-R.); psoto@sjdhospitalbarcelona.org (P.S.-U.); 3 Department of Psychiatry and Mental Health, Hospital Clínico San Carlos, Madrid, Calle del Prof Martín Lagos, s/n, 28040 Madrid, Spain; isabelmsc.is@gmail.com (I.d.l.M.S.-C.); biancbor@ucm.es (B.B.); marinadiaz.marsa@salud.madrid.org (M.D.-M.); 4 Department of Psychiatry and Mental Health, Hospital Universitario de Bellvitge-IDIBELL and CIBEROBN, Barcelona, Carrer Feixa Llarga s/n, 08907 Hospitalet del Llobregat, Barcelona, Spain; isasanchez@bellvitgehospital.cat (I.S.-D.); ffernandez@bellvitgehospital.cat (F.F.-A.)

**Keywords:** eating disorders, virtual reality body exposure, fear of gaining weight, body image disturbances, anorexia nervosa

## Abstract

In vivo body exposure therapy is considered an effective and suitable intervention to help patients with anorexia nervosa (AN) reduce their body image disturbances (BIDs). However, these interventions have notable limitations and cannot effectively reproduce certain fears usually found in AN, such as the fear of gaining weight (FGW). The latest developments in virtual reality (VR) technology and embodiment-based procedures could overcome these limitations and allow AN patients to confront their FGW and BIDs. This study aimed to provide further evidence of the efficacy of an enhanced (by means of embodiment) VR-based body exposure therapy for the treatment of AN. Thirty-five AN patients (16 in the experimental group, 19 in the control group) participated in the study. FGW, BIDs, and other body-related and ED measures were assessed before and after the intervention and three months later. The experimental group received treatment as usual (TAU) and five additional sessions of VR-based body exposure therapy, while the control group received only TAU. After the intervention, ED symptoms were clearly reduced in both groups, with most of the changes being more noticeable in the experimental group. Specifically, after the intervention and at follow-up, significant group differences were found in the FGW and BIDs, with the experimental group showing significantly lower values than the control group. The current study provides new insights and encouraging findings in the field of exposure-based therapies in AN. VR technology might improve research and clinical practice in AN by providing new tools to help patients confront their core fears (i.e., food- or weight-related cues) and improve their emotional, cognitive, and behavioral responses to their body image.

## 1. Introduction

Eating disorders (ED) are severe disorders characterized by dysfunctional patterns in eating or weight-control behaviors that affect the individual’s physical and mental health [1]. Anorexia nervosa (AN) is one of the most severe types of EDs, affecting approximately 1–4% of European women and 0.3–0.7% of men [2]. It has higher mortality rates than other EDs and psychopathologies, with one in five deaths resulting from suicide [3,4]. Furthermore, AN is increasingly being diagnosed in younger patients [5,6], with a typical age of onset of around 14 and 19 years [7,8]. Some studies report that approximately 40% of new AN cases are diagnosed during early adolescence or adolescence [3,5].

One of the main core fears among individuals with AN is the fear of gaining weight (FGW). The FGW is an overconcern and an extreme fear of the possibility of gaining weight in the whole body or in some specific body parts, even at a significantly low weight [1,9]. Individuals with AN may also display dysfunctional behaviors that aim to avoid weight gain by any means possible (e.g., by drastically reducing food intake, vomiting, using laxatives and diuretics, or doing intense exercises, etc.) [1]. 

The FGW level reported by AN patients may vary during the disorder or during treatment. For instance, some patients experience a significant increase in FGW levels after a small weight restoration in an ED inpatient unit. This, a small degree of positive weight gain activates a series of catastrophic beliefs based on continuous and uncontrollable weight gain expectations, which in turn lead to an exponential increase in FGW levels [10]. A recent study using network analyses found that most ED symptoms/pathology can be explained not by the unique effect of gaining weight, but by the negative associated consequences that patients usually report [11]. Based on this last assumption, it has been proposed that the extinction of the FGW in AN does not solely occur via a progressive habituation process towards weight recovery, but also via mechanisms of the inhibitory learning systems [10]. Thus, the FGW construct can be also understood as a feared outcome/consequence, and individuals presenting a great FGW can learn new healthy associations with their own weight and silhouette when their threatening weight-related expectations are not met (e.g., if I increase my weight… I will not resist it, people are going to laugh at me or tell me that I am fat, etc.). 

Several studies have suggested a direct relationship between the FGW and greater ED symptomatology [12,13], indicating that the FGW is a core mechanism underlying AN symptomatology. Indeed, recent studies have found that the FGW is directly associated with overvaluing the body and feeling guilty after overeating [14], with the FGW being the strongest predictor of ED symptomatology (e.g., dietary restraint) in AN patients [15].

Individuals with AN also present body image disturbances (BIDs), which are cognitive and emotional disturbances in the way in which one’s body weight or shape is experienced [16] (Legenbauer, Thiemann, and Vocks, 2014). Body image distortion (the perceptual component) and body image dissatisfaction (the affective component) are the most studied BIDs [17,18]. Individuals with EDs, particularly AN, usually display a series of avoidance behaviors and negative checking strategies towards their own body, which lead to a dysfunctional body-related attention or attentional bias (AB; [19,20]). Previous studies suggest that body-related AB may be an important factor maintaining BIDs in patients with EDs and healthy individuals [20,21,22]. For instance, individuals who are very dissatisfied with some of their body areas (i.e., the thighs) tend to focus their attention repeatedly and constantly on those specific body areas, which may trigger and reinforce a series of negative body-related beliefs (e.g., my thighs are getting fatter) that lead to higher body dissatisfaction levels [20]. Furthermore, previous research has found that body-related AB seems to be especially pronounced in adolescent patients with restrictive AN [23]. 

Several studies have suggested the necessity to implement specific tailored interventions that aim to decrease fears and other body-related disturbances. This has led to improvements in evidence-based therapies (i.e., cognitive behavioral therapy, CBT) in AN and other EDs [10,24,25,26,27,28]. Exposure-based therapies provide a good example of interventions targeting specific food- and body-related fears, such as the FGW in AN [27]. Previous studies have presented promising results for exposure-based therapies conducted as part of CBT or as a separate intervention [28,29,30]. 

Among the exposure-based procedures using body image cues, mirror exposure therapy (MET) has been widely used. This usually involves the patient systematically observing their body or specific body parts for a certain amount of time [31,32] in a neutral descriptive way (non-judgmental MET; [33]), a positive descriptive way (cognitive dissonance-based MET; [34]), or in a freely descriptive way, with the individual freely expressing their emotions and thoughts about their body (pure MET; [35]). Pure MET has been shown to be the most effective in reducing body dissatisfaction [35]. 

MET has already been successfully used as a complement to AN treatment [36], reducing negative body-related emotions and cognitions through a habituation process [37]. However, more research is needed with randomized controlled clinical trials involving larger sample sizes and follow-up results [29]. In-vivo mirror and body exposure techniques may be difficult to implement in severe cases of AN; due to the risk of eliciting habituation toward an extremely low weight (or very skinny body shape), they may be contraindicated in these patients [38,39]. Other studies emphasize the need to implement body shape interventions during intensive hospitalization treatment programs in AN [40]. The application of new technologies such as virtual reality (VR) could provide innovative solutions to some of the previous problems.

VR is a transformative technology that has been widely used in the last decade to improve exposure-based therapies. It is associated with improved treatment adherence and acceptance rates compared to in vivo exposure therapy [41]. VR technology can be also used to create highly realistic simulations of everyday life situations associated with the patients’ body or weight concerns, as well as three-dimensional (3D) avatars that reproduce the patients’ silhouettes based on their own body size, skin, height, and clothes [42,43].

Furthermore, VR technology now allows individuals to be “embodied” in a virtual avatar, or in other words, to perceive and regard a virtual body as their own real body, experiencing the illusion of owning the virtual body [44] in what is known as full body illusion (FBI). FBI is defined as the subjective experience in which individuals regard an artificial body as their own body by combining different types of information (visual, acoustic, proprioceptive, vestibular, etc.) into different multisensory representations [45,46]. One of the most studied body ownership paradigms is the rubber hand illusion [47]. 

The continuing improvements in VR embodiment-based techniques have produced new applications for improving health-related issues in a transdisciplinary research field known as embodied medicine [48,49]. Previous research on body image has already demonstrated that VR embodiment-based procedures can modify and even improve BIDs [40]. For instance, these techniques can change the perception of the whole body or specific body parts among healthy participants [46,50,51] and patients with EDs [52,53,54].

While most of the in-vivo body exposure-based therapies, such as MET and VR-based interventions, have focused on treating BIDs, little information is available about the use of body exposure techniques, with or without VR, to treat the FGW. Imaginal exposure might help patients with AN confront their core FGW, which is impractical to do with other techniques. Two studies have presented very encouranging results for imaginal exposure: a case report [55] and a clinical study with a large sample of patients with EDs [56]. The latter study reported that after conducting four weekly sessions of online and self-guided imaginal exposure, ED symptoms and ED-related fears significantly decreased after the therapy and at the six-month follow-up [56]. That study provided the first evidence of the effectiveness and usefulness of imaginal exposure techniques in reducing fears and ED-related symptomatology. However, some patients who undergo imaginal exposure might report difficulties by achieving or maintaining a visualization over time, and may also display avoidance-based strategies while they imagine their most feared stimulus during visualization (e.g., visualizing a progressive weight gain). VR technology can overcome these limitations, since it does not rely on the visualization ability of the patient (e.g., while patients own their real-size virtual body, they can experience a weight gain in a more vivid way). In addition, this technology can significantly reduce avoidance-based behaviors, for instance, by using eye-tracking (ET) devices on the VR-HMD to control the patient’s gaze patterns towards their own body. 

To date, there has been only one case report discussing the use of a VR embodiment-based paradigm in targeting the FGW in AN. In that case report, a patient with AN underwent a structured exposure intervention owning a virtual body that simulated their own body, leading to an increase in the BMI until a healthy BMI was obtained. In that report, there was a decrease in the FGW and related ED symptomatology after five sessions of VR body exposure therapy [54].

Based on these promising results, the current study aimed to go further and provide additional evidence of the usefulness of a VR body exposure therapy in AN (hereinafter referred to as AN-VR-BE) in directly targeting the FGW and other body-related disturbances using a larger sample and a controlled experimental design. In particular, the current study aimed to assess the effectiveness of AN-VR-BE through a randomized controlled clinical trial (clinicaltrials.gov (accessed on 1 January 2021), NCT 04028635), in which patients with AN were evaluated before and after the intervention, and three months later. The starting hypothesis was that if a component of body exposure through VR (intensified through an illusion of virtual body ownership) was added to the usual treatment for AN (experimental group), the treatment would be more effective than usual treatment only (TAU, the control group). It was expected that after comparing measures before and after the treatment, and three months later, the experimental group (AN-VR-BE + TAU) would show a significant increase in BMI values alongside significant reductions in FGW levels, other ED symptomatology, and body-related AB when compared to the control group (TAU).

## 2. Materials and Methods

### 2.1. Clinical Sample 

Thirty-five adolescent and adult patients with AN participated in the study. All the patients were randomly assigned to the two groups: 16 patients were assigned to the experimental group and 19 patients to the control group. Most of the patients were women, with only four men in the sample who were split equally between the two groups. All the adolescent patients received treatment at the ED Unit of Hospital Sant Joan de Déu of Barcelona. Adult patients received treatment at the ED Units of the Hospital of Bellvitge and Hospital Clínico San Carlos of Madrid. All patients were diagnosed in their respective ED Units. 

All patients diagnosed with AN using the DSM-5 criteria [1], aged 13 years or over, with a BMI < 19, and able to understand and read Spanish were invited to participate in the study. One patient who did meet all the DSM-5 criteria for AN except for two also participated in the study. The exclusion criteria were serious mental disorders with psychotic or manic symptoms (e.g., psychotic disorders, bipolar disorders, etc.), sensory complications that would prevent exposure (e.g., visual, tactile, or auditory deficits), epilepsy, and other medical conditions (such as pregnancy and clinical cardiac arrhythmia). 

### 2.2. Measures

#### 2.2.1. Pre-Post Assessment Measures

##### Visual Analog Scales (VAS)

- Self-reported FBI levels were assessed within the VR environment using a VAS to determine the extent to which the patients felt the virtual body was their own body on a scale ranging from 0 (not at all) to 100 (completely).
- Self-reported FGW levels were assessed within the VR environment using a VAS to determine the extent to which the patients were afraid of gaining weight while owning the virtual body on a scale ranging from 0 (not at all) to 100 (completely).
- Self-reported anxiety levels were assessed within the VR environment using a VAS to determine the extent to which the patients were anxious about their own body while owning the virtual body on a scale ranging from 0 (not at all) to 100 (completely).

##### AB Measures

Body-related AB was measured by determining the patient’s visual fixation on their own body. Visual fixation has been defined as an involuntary act of maintaining the gaze on a specific spot, at least, for 100–200 ms [57]. In this study, the duration established to define a fixation was 100 ms. The two main groups of areas of interest (AOIs) of the body were defined based on the Physical Appearance State and Trait Anxiety Scale (PASTAS). Specifically, the weight-related AOIs (W-AOIs) were the stomach, hips, waist, thighs, and legs, which were individually drawn onto a two-dimensional frontal view picture of the female or male avatar. The remaining body areas were also drawn onto the same image and were labeled as non-weight-related AOIs (NW-AOIs). 

Visual fixation was estimated from the number of fixations (i.e., the sum of the available fixations on the specified AOI group) and the total fixation time (i.e., the sum of the fixation duration for the specified AOI group in milliseconds). Both measures are considered reliable and provide a continuous way of assessing body-related AB. They have been widely applied in previous studies using ET technology [58]. 

#### 2.2.2. Pre-Assessment, Post-Assessment, and Three Months Follow-Up Measures

Body-related measures and other ED clinical symptomatology.

- The BMI was used as a common indicator of body fatness to assess changes in weight in the pre-, post-, and follow-up assessments. The BMI was calculated by dividing the patient’s weight (in kilograms) by the square of their height (in meters).
- Eating Disorder Inventory 3 (EDI-3) [59]. The EDI-3 is a widely used self-report and standardized measure to assess symptomatology and psychological features relevant to the development and maintenance of EDs. The third version (EDI-3) includes 91 items classified into 12 scales, with a six-point Likert scale for each answer ranging from 0 (never) to 5 (always). The EDI-3 questionnaire and materials have been translated into Spanish and validated [60]. This Spanish version presents robust validity and reliability indices (like the original version), with a Cronbach’s alpha ranging from 0.74 to 0.96. In the current study, we only used the Body Dissatisfaction (EDI-BD) and Drive for Thinness (EDI-DT) scales. The EDI-BD scale, with 10 items, measures the negative subjective attitude or evaluation of one’s body or specific body areas, including their shape, weight, and fitness. The EDI-DT assesses the strong desire to have a thinner body and to lose weight, as reflected by the intense concerns about the body shape or weight, the diet and the FGW. The Cronbach’s alpha values in this study were 0.825 for the EDI-BD scale and 0.84 for the EDI-DT scale. The fourth item of the EDI-DT scale (i.e., “I am terrified about gaining weight”) was also used as an independent measure to determine the FGW in the pre-, post-, and follow-up assessments.
- Physical Appearance State and Trait Anxiety Scale (PASTAS) [58]. The PASTAS is a reliable and valid measure for the assessment of trait and state body image anxiety. Patients had to rate, on a five-point scale ranging from 0 (never) to 5 (always), if they felt anxious or nervous about their physical appearance, including any tension, negative thoughts, and physiological responses. Although the questionnaire comprises two scales (weight-related and non-weight-related scales) that measure anxiety for 16 body areas, only the eight-item Weight Scale was used in this study. The scale presents good reliability and reliability indices [61]. In the current study, Cronbach’s alpha was 0.817.
- The Body Image Assessment Scale-Body Dimensions (BIAS-BD) [62] was used to assess the perceptual and emotional components of BIDs. This test assesses the discrepancy between the perceived body size and the self-determined ideal body size (to measure body dissatisfaction). Furthermore, it also reveals the discrepancy between the perceived body size and the real body size (to measure body distortion). The scale presents a range of 17 silhouettes, with different versions for women and men. The two test–retest A–B versions were used in the pre-assessment (version A), post-assessment (version B), and follow-up assessment (version A again). Patients had to select the silhouette that they perceived to be the most similar to their own and the silhouette that they would like to have. Finally, the researchers also selected the real silhouette based on the patient’s BMI.
- The Body Appreciation Scale (BAS) [63] was used to assess positive attitudes towards one’s own body. This 12-item scale was translated into Spanish by Jáuregui-Lobera and Bolaños-Ríos [64]. The scale measures a single dimension of a positive body image, which includes having a positive opinion and accepting one’s physical features, the adoption of healthy behaviors with regards to one’s body, and the rejection of dangerous body ideals usually displayed in social media. The BAS items are rated on a five-point Likert scale that is averaged to obtain a final score of body appreciation, which ranges from 0 to 60, with higher values suggesting greater body appreciation.

### 2.3. Technical Features

#### 2.3.1. Hardware

All the ED Units that participated in the study had a complete VR package and powerful VR-ready computers equipped with high-end CPU processors (i.e., Intel i7 processors) and powerful graphic cards (i.e., Nvidia GTX 1080) to fluently run 3D immersive virtual environments. The whole VR system included head-mounted displays (HTC-VIVE HMD), two different VR controllers that the patient held in their hands, and three additional body trackers that were attached to the feet and waist of the patients. All the equipment, including the VR-HMD, controllers, and body trackers, was used to capture the whole body movements of the patients (full-body tracking system). Furthermore, two HTC-VIVE base stations were placed at a considerable height, approximately two meters off the ground (using tripods), and diagonally positioned at opposite corners of the doctor’s office, to create a minimum play area of 2 m × 1.5 m (6 ft 6 in × 5 ft). 

Finally, a second headset with incorporated ET technology (FOVE VR-HMD) was used to detect eye movement while the patients were immersed in the VR environment before and after the intervention. The FOVE VR-HMD uses 120 Hz infrared ET sensors, with a resolution of 2560 × 1440 pixels and a spatial accuracy of less than one degree.

#### 2.3.2. Software

The female and male avatars were initially designed using the software Blender v. 2.78, which is particularly useful for designing specific details of the body, such as the clothes or the skin, and for creating a complete skeleton of the virtual avatar that can reproduce realistic movements using a full-body tracking system. Both the female and male avatars wore standard clothes (a white t-shirt, blue jeans, and black trainers). The hair was covered by a gray hat to reduce any influence of hairstyle on each patient. The avatars also wore an HMD like the patients. Finally, the height could be easily adjusted based on the patient’s height. 

The Unity 3D 5.6.1 (Unity Technologies) software was used to design the VR room, develop the programming code, and incorporate the virtual avatars within. The virtual environment consisted of a unique room without any furniture, except for a large mirror on the wall placed 1.5 m in front of the patient. The patients could see their whole body reflected in the mirror, even when they were moving.

### 2.4. Procedure

#### 2.4.1. Overview

The ethics committees of the University of Barcelona (Institutional Review Board IRB00003099) and the three hospitals that participated in this study reviewed and approved this study. Furthermore, the study protocol and reporting was conducted in accordance with the statement of the Consolidated Standards of Reporting Trials (CONSORT) [65]. All the patients accepted into each of the ED units received a complete psychological, nutritional, and medical assessment, and were diagnosed by clinical psychologists and psychiatrists. All the ED Units used, at least, a non-structured clinical interview to assess the fulfillment of the DSM-5 criteria, and, in two of them, the Mini-International Neuropsychiatric Interview (MINI; [66]) was also used by trained clinicians. 

All the ED Units offered three different ED treatment programs: an outpatient program for patients with good treatment compliance and lower biopsychological risk, a day-patient treatment for those whose weight restoration and eating behaviors were not improving, and finally an intensive 24-h hospitalization program for patients with a high biopsychological risk (i.e., very low weight). 

The current study focused only on patients who attended the day patient program. These patients underwent a multidisciplinary treatment that consisted of CBT conducted individually and in groups, nutritional rehabilitation that aimed to improve eating patterns and increase weight, and group counseling for their relatives. Individual CBT consisted of a minimum of one session per week, usually two sessions per week, for both adolescent and adult patients, each lasting 45–60 min. Adolescent patients attended the inpatient treatment unit for 11 h a day (sleeping at home). For a more detailed description, see Serrano-Troncoso et al. [67]. The adult patients attended the ED Unit for over 6 h a day. Furthermore, adult patients diagnosed with a borderline personality disorder were also treated in a specialized unit for approximately 4 h a day.

All patients were enrolled in the study at similar times after the beginning of the day-patient program treatment. Once the study began, all the clinicians from the ED units who had direct contact with the patients checked whether they met the inclusion and exclusion criteria. During the first week, patients were informed of the possibility of participating in the study. They were then enrolled during the second or third week, while the same clinicians obtained written informed consent from all the patients included in the study (in the case of the adolescent patients, informed consent was also obtained from their parents or legal guardians). All the sessions were carried out by a psychologist with previous clinical experience and a co-therapist who helped provide additional support on some of the tasks conducted during the treatment.

**Figure 1:**
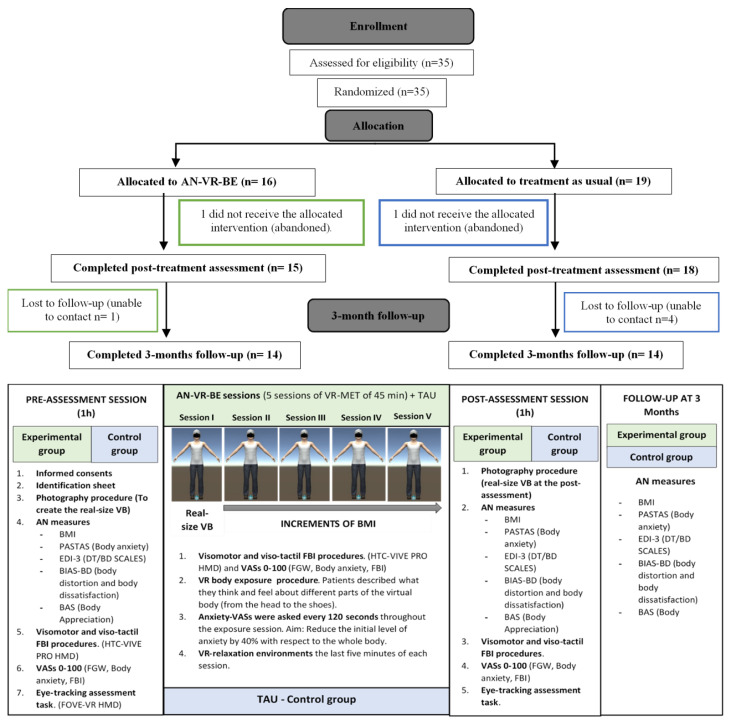
Participant flow throughout the study and the experimental design of the study.

As shown in Figure 1, all the patients were assessed three times: in the pre-assessment session, after five weeks in the post-assessment session, and at follow-up three months later. Furthermore, all the patients were randomly assigned to one of the two groups: the experimental group receiving VR-based body exposure alongside the usual CBT or the control group receiving the usual treatment only. 

Randomization of the sample was conducted using the AleatorMetod.xls software [68] (Ramos-Alvarez, 2005) before the start of the study. 

#### 2.4.2. Pre-Assessment, Post-Assessment, and Follow-Up Sessions

The pre-assessment session lasted approximately one hour. Since the procedures conducted in the pre-assessment (photography, visuomotor, and visuo-tactile stimulations and ET assessment task procedures) have been described in a previous study by our group, this section has been summarized. For a detailed description, see [69]. 

In the first session, any doubts or questions that the patients had about the first session or the whole treatment were addressed. The patients were also informed about the confidentiality of the data and were provided with a specific identification code. 

**Figure 2:**
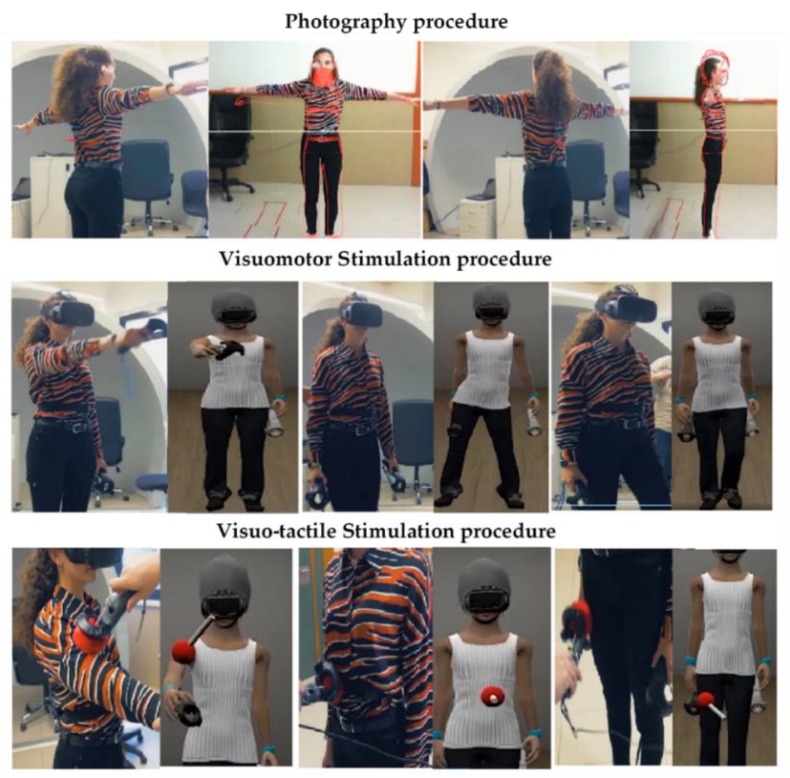
Visuomotor and visuo-tactile stimulations conducted in the pre-assessment and post-assessment sessions and in each treatment session.

The first step was to create the virtual avatar. A photograph of the patient was taken using a frontal and lateral perspectives, with the silhouette of the real-size avatar matched to the pictures. While the virtual avatar was being created, the patients had to complete all the pre-assessment questionnaires. The patients were then immersed in the VR environment, in which they underwent visuomotor and visuo-tactile stimulation procedures, adapted from previous studies [69,70,71,72], to elicit FBI over the virtual body. Both procedures lasted three minutes, and at the end, the FBI, body-related anxiety and the FGW that they were experiencing at that moment were assessed using the VAS. For an illustrative description of the procedure, see Figure 2.

The last step consisted of conducting the ET task. The previous headset (HTC-VIVE HMD) was replaced with the new ET FOVE VR headset. After a brief calibration and validation procedure, the patients were exposed to the same virtual environment as before. They were asked to observe their virtual body reflected in the mirror, while their spontaneus gaze was tracked for 30 s, as done in previous studies [73,74]. As a cover story, they were told that the full-body tracking system (i.e., body trackers and controllers) was being recalibrated and that it was important that they remained still during the process. 

The same procedures with VR and ET were conducted in the post-assessment session. The BMI and silhouette of the virtual body were updated in the patients who had increased/decreased their real BMI values in the post-assessment session. Finally, at the follow-up assessment session, the VR and ET procedures were not conducted, since most of the patients had been referred to outpatient services and were no longer being treated in the ED unit. Thus, the clinicians from the ED units directly collected or sent by email the paper-based questionnaires for the follow-up measures.

#### 2.4.3. VR Body Exposure Sessions (Experimental Group)

Before starting the treatment, the clinicians responsible for each patient proposed a healthy BMI target for the patients to reach. The target BMI was calculated by considering the patient’s age and their BMI prior to the onset of the disorder. Therefore, the target BMI was not the same for all the patients, but tailored to each case.

Exposure sessions were conducted weekly, face to face, lasting approximately 60 min. In the first session, the patients were exposed to a virtual body with their real-size silhouette and BMI (Figure 1). Then, small BMI increases were applied to the virtual body over successive exposure sessions, until the healthy BMI target was finally reached. For instance, an adult patient with a BMI of 17 kg/m^2^ in the first session who was aiming to reach a healthy BMI of 19 kg/m^2^ had to undergo small BMI increases of 0.5 kg/m^2^ over the four subsequent sessions.

Before starting each body exposure session, the clinicians answered any questions that the patients might have had about the current or past sessions. Once in the VR environment, each session was initiated by conducting the visuomotor and visuo-tactile stimulation procedures to induce the FBI over the virtual body. The VAS assessing the FBI level was also applied. The body exposure task began by asking the patients to orally report their thoughts and feelings about most of the body parts of their virtual body (starting from the head, shoulders, arms, hands, chest, stomach, waist, hips, thighs, lower legs, and then the feet). This task allowed the patients to spread their visual attention across the whole virtual body. 

At the same time, their anxiety levels were monitored, using a VAS, every two minutes during the exposure session. The body exposure session was ended once the anxiety levels had been reduced by 40% compared to the initial anxiety level or once the duration of the session had elapsed. The last 5 min of the session were dedicated to helping the patients reduce their anxiety or any other sort of discomfort that they might have experienced during the body exposure task. The patients were immersed in relaxing VR environments (i.e., waterfalls, forests, or beaches) for debriefing. 

The aim of the intervention was to continue to the next item in the hierarchy (i.e., a BMI increase). However, for the patients who had not reduced their anxiety level by 40% during the session, they were exposed again to the same avatar in the following session. 

### 2.5. Statistical Analyses

The Open Gaze and Mouse Analyzer (OGAMA) software was used to prepare the ET data. For further details on this procedure, see [69]. 

All the subsequent statistical analyses were performed with SPSS version 24. First, Fisher’s exact test for categorical variables and independent-samples *t*-test for continuous variables were conducted to assess whether there were any significant group differences in any of the demographic and clinical measures assessed at baseline/pre-assessment. Then, mixed between (group)–within (pre-post-follow-up assessment times) analyses of variance (ANOVA) were conducted for the BMI and all the ED measures (primary measures). Furthermore, mixed between (group)–within (pre-post-assessment times) analyses of variance (ANOVA) were conducted for AB measures and the VAS scores (secondary measures). 

Regarding the assumptions of the tests, homogeneity of variance was met for most of the variables (*p* > 0.05), as demonstrated by Levene’s test. The sphericity test (*p* > 0.05) was also met for almost all the variables. Although some of the variables (e.g., the BMI, EDI-DT-FGW, and some of the VAS scores) were not normally distributed, as determined by the Shapiro–Wilk test, it was decided to run the tests anyway, since ANOVA is considered a reasonably robust test to deviations from normality [75]. Finally, two outliers were detected in the BMI, one of which affected the other measures as well, which was identified by the inspection of a boxplot. After conducting the analyses with and without the outliers, it was decided that they would be kept in the analyses, since the results did not vary significantly. 

## 3. Results

### 3.1. Descriptive Results

The patients had a mean age of 18.63 years (SD = 6.78, age range: 13–44 years), a mean BMI of 17.48 kg/m^2^ (SD = 1.12, BMI range: 14.25–18.95 kg/m^2^), and were mainly female (31/35, 88.57%). Moreover, 60% of them were adolescents (21/35), while 40% were adults (14/35). The other demographic and clinical characteristics of the groups are shown in Table 1. 

Fisher’s exact test and independent-samples *t*-test showed that there were no significant differences between the groups at baseline in any of the demographic and clinical variables, including medication, main diagnosis, and the number of comorbid diagnoses. Furthermore, independent-samples *t*-test analyses showed that there were no statistically significant group differences (*p* > 0.05) in the pre-assessment session, in any of the clinical measures.

**Figure 3:**
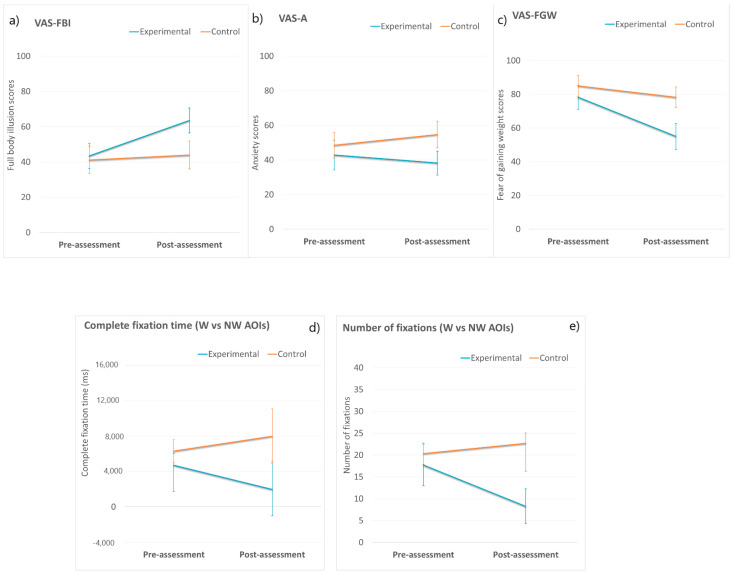
Means of the experimental and control groups in the two assessment conditions (pre-assessment and post-assessment) in Full Body Illusion (**a**), Anxiety (**b**), Fear of Gaining Weight (**c**), Complete fixation Time (**d**) and Number of Fixations (**e**). Error bars represent standard errors of the mean. Note: Visual Analog Scales (VAS), Full Body Illusion (FBI), Anxiety (A), Fear of Gaining Weight (FGW), Weight (W) and Non-Weight (NW) Areas of Interest (AOIs). * *p* values < 0.05.

**Figure 4:**
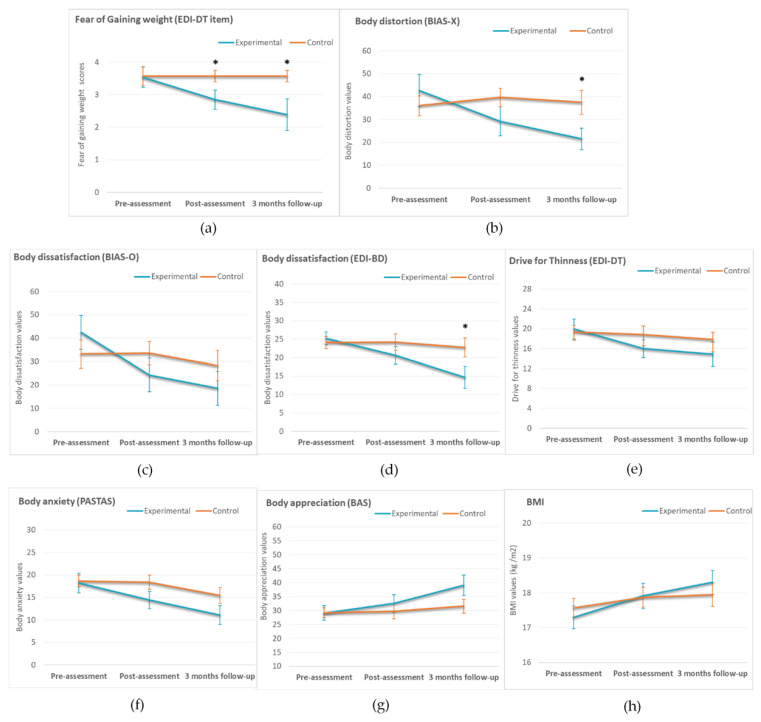
Means of the experimental and control groups in the three assessment conditions (pre-assessment, post-assessment, three months follow-up) in Fear of Gaining Weight (**a**), body distortion (**b**), body dissatisfaction as measured by the EDI-3 (**c**) and the BIAS-BD (**d**), Drive for thinness (**e**), body anxiety (**f**) body appreciation (**g**) and Body mass index (**h**) Complete fixation Time (3d) and Number of Fixations (3e). Error bars represent standard errors of the mean. Note: BMI = Body mass index. * *p* values < 0.05.

Figure 3 and Figure 4 show the means and standard errors of the mean of all the clinical measures for the experimental and control groups at the different assessment times.

### 3.2. FBI, Body Anxiety and FGW VASs

Two-way mixed ANOVA was conducted for FBI, body anxiety, and the FGW using the VAS scores (see Figure 3a–c, respectively). The results indicated a lack of statistically significant interactions between the group and assessment time (*p* > 0.05) for FBI (VAS-FBI) and body anxiety (VAS-A). However, there was a main effect of time on FBI (*F* (2, 96) = 3.297, *p* = 0.044, partial η^2^ = 0.147). Overall, despite no significant group differences, individuals in the experimental group reported higher FBI levels in the post-assessment session than in the pre-assessment session, while the control group reported similar FBI levels before and after the intervention.

Regarding the FGW, there was a marginally significant interaction between group and assessment time (*F* (1, 26) = 3895, *p* = 0.059, η^2^ = 0.130). Follow-up analyses showed that the FGW scores did not differ between the two groups (*p* > 0.05) in the pre-assessment, but there were significant group differences in the post-assessment (*F* (1, 26) = 5611, *p* = 0.028, partial η^2^ = 0.173), with a high effect size according to Cohen (1998). The experimental group reported significantly lower FGW values compared to the control group (*MD* = 23.21, *SE* = ±9.94).

### 3.3. Body-Related Attentional Bias

Two-way mixed ANOVA was conducted for both attentional bias measures. The results indicated a lack of statistically significant interactions between the group and assessment time for the total fixation time (*F* (1, 21) = 1025, *p* = 0.323, partial η^2^ = 0.047) and the number of fixations (*F* (1, 21) = 2782, *p* = 0.110, partial η^2^ = 0.117).

Although there were no statistically significant group*time interactions, there was a clear trend at post-assessment in which patients in the experimental group showed lower AB towards W-AOIs than those in the control group, who showed higher AB towards W-AOIs (total fixation time: MD = 6011, SE = ±3703; number of fixations: MD = 14.39, SE = ±7.69). For illustrations, see Figure 3d,e.

### 3.4. ED Measures (Pre-Assessment, Post-Assessment, and Three-Month Follow-Up)

Two-way mixed ANOVA was conducted for all the ED measures. There were significant interactions between group and assessment time for body distortion (*F* (2, 50) = 7.763, *p* = 0.002, partial η^2^ = 0.218), body dissatisfaction assessed with the BIAS-BD questionnaire (*F* (2, 52) = 5.084, *p* = 0.033, partial η^2^ = 0.123), and body dissatisfaction assessed with the EDI-BD (*F* (2, 52) = 4.425, *p* = 0.017, partial η^2^ = 0.145). Furthermore, marginally significant interactions between group and time were found for body appreciation (*F* (2, 52) = 3.065, *p* = 0.057, partial η^2^ = 0.104) and FGW, as assessed by the EDI-DT-FGW item (*F* (2, 50) = 2.671, *p* = 0.078, partial η^2^ = 0.097). Follow-up analyses in all these measures showed that there were significant group differences on FGW at the post-assessment (*F* (1, 25) = 4.626, *p* = 0.041, partial η^2^ =.156), and there were significant group differences at follow-up for body distortion (*F* (1, 25) = 5.264, *p* = 0.030, partial η^2^ = 0.174), body dissatisfaction assessed with the EDI-BD (*F* (1, 26) = 4.320, *p* = 0.048, partial η^2^ = 0.142), and FGW (*F* (1, 25) = 5.584, *p* = 0.026, partial η^2^ = 0.186). As shown in Figure 4a,b,d, after the intervention and especially at follow-up, the experimental group reported significantly lower levels of FGW, body distortion, and body dissatisfaction compared to the control group. Despite the experimental group showed a more pronounced descrease in body dissatisfaction scores assessed with the BIAS-BD (Figure 4c), and a more pronounced increase in body appreciation values (Figure 4g) compared to the control group, group differences did not reach statistical significance (*p* > 0.05) in the post-assessment session or at follow-up.

Finally, there were no statistically significant group*time interactions (*p* > 0.05) for any of the other ED measures assessed (BMI, drive for thinness and body anxiety). However, there was a main effect of assessment condition on BMI (*F* (1, 60) = 11.407, *p* ≤ 0.001, partial η^2^ = 0.275), body anxiety (*F* (1, 52) = 6573, *p* = 0.003, partial η^2^ = 0.202), and drive for thinness (*F* (1, 52) = 3.508, *p* = 0.037, partial η^2^ = 0.119). Post-hoc tests (pairwise comparisons) were conducted, and results showed that regardless of the group, there were significant differences (*p* < 0.05) between pre- and post-assessment in BMI (*MD* = −0.45, *SE* = 1.12, *p* = 0.002), and between pre-assessment and follow-up in BMI (*MD* = −0.69, *SE* = 1.66, *p* = 0.001) and body anxiety levels (*MD* = 5.18, *SE* = 1.54, *p* = 0.007). There were also marginally significant differences between pre-assessment and follow-up (*MD* = 2.28, *SE* = 1.35, *p* = 0.068) for drive for thinness. As can be seen in Figure 4e,f,h, regardless of the group, there was a continuous decrease in symptomatology in the post-assessment and at follow-up for BMI, body anxiety, and drive for thinness. Again, this decrease seemed to be more pronounced in the experimental group.

### 3.5. Age as a Predictor of a Worse Treatment Outcome at Post-Assessment

Based on the descriptive data, adult patients tended to report greater ED symptomatology at post-assessment, including a greater FGW, than the adolescent patients. This was especially noticed in the experimental group, suggesting that the VR intervention might be more effective in young patients than in adults. Thus, post-hoc simple linear regression analyses were run in each group separately to assess whether age was a significant predictor of a worse clinical outcome.

In the control group, linear regression analyses showed that age did not significantly (*p* > 0.05) predict greater ED symptomatology, including the FGW, in any of the measures assessed. However, a higher age did significantly predict lower values of FBI after the intervention (*F* (1, 14) = 7.191, *p* = 0.019) that accounted for 35.6% of the explained variability.

**Figure 5:**
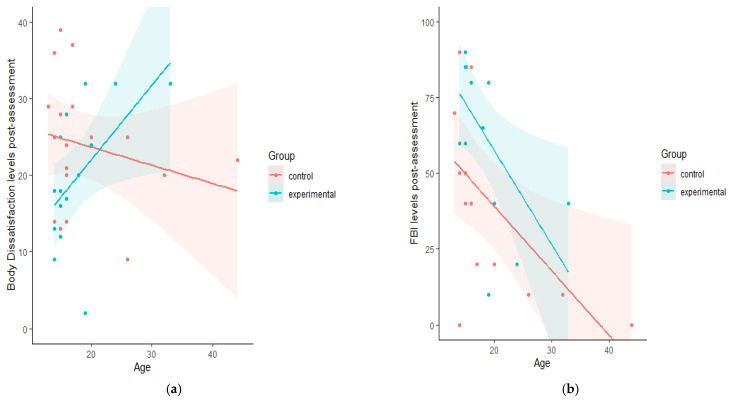
Scatterplots assessing the linear relation between Body Dissatisfaction at the post-assessment, as measured by EDI-3, and age (**a**). And the relation between Full-Body Illusion (FBI) levels at the post-assessment and age (**b**).

In the experimental group, age was a significant predictor of higher values of body dissatisfaction assessed with the EDI-3 (*F* (1, 14) = 5.682, *p* = 0.033), which accounted for 30.4% of the explained variation. Furthermore, a higher age also significantly predicted lower FBI levels (*F* (1, 13) = 6.678, *p* = 0.024) that accounted for 35.8% of the explained variation. For an illustration see Figure 5a,b.

## 4. Discussion

As previously stated, AN is one of the most serious EDs, with very worrying rates of mortality compared to other disorders [76] and an increasingly higher prevalence of diagnoses in early adolescence [3,5]. Thus, focusing on new interventions that aim to reduce the core fears (such as the FGW) and other body-related concerns is essential to improve evidence-based therapy in AN. The current study provides a new insight and encouraging findings in the field of exposure-based therapies in AN. Based on our results, the addition of body exposure therapy through VR (experimental group) to the usual treatment for AN was more effective than usual treatment only (control group), particularly for the FGW and BIDs. This intervention was effective in reducing FGW levels, as observed by significant group differences in the post-assessment and follow-up, in which the experimental group showed a significantly higher reduction in FGW scores than the control group. Furthermore, this intervention was also effective in reducing BIDs. Indeed, after the intervention and especially at follow-up, the experimental group reported significantly lower levels of body distortion and body dissatisfaction than the control group. Furthermore, similar tendencies (although not significant) were found among all the other measures, in which the experimental group showed a more pronounced decrease in ED symptomatology and a higher increase in the BMI and body appreciation values compared to the control group. These tendencies were maintained at the three-month follow-up. Finally, preliminary evidence suggested that age was a significant predictor of a worse clinical outcome after the intervention.

The current study is the first to develop a tailored, controlled, and empirically validated VR-based body exposure intervention to help patients with AN confront their core fear, the FGW. The current results also provide further evidence supporting the findings from a preliminary case report also conducted by our group [54]. Furthermore, our results suggest that there was a continuous weight restoration in both groups after the intervention and after three months, being slightly higher in the experimental group. These results are important, since weight restoration is considered a robust predictor of remission from AN and is necessary for recovery [77].

Our results are also in line with those of previous studies that used imaginal exposure to reduce the FGW and other ED symptomatology in patients with EDs [55,56]. As previously noted, virtual or imaginal exposure-based therapies are an innovative and interesting way of helping patients with AN confront situations or fears that is difficult to achieve with other techniques. For instance, these techniques can be used to modify the content of the negative consequences associated with the FGW that patients with AN usually report.

The clinical impact of the FGW and its associated negative consequences in patients with AN are well-known, being directly associated with other ED symptoms [11,14]. Indeed, several studies have claimed that tailored behavioral interventions targeting the FGW in AN are effective in reducing other ED symptoms [10,26,27,28]. Our results might provide evidence supporting this approach. Even though significant group differences were found for only a few measures (BIDs), there was a more pronounced reduction in ED symptomatology in the experimental group than in the control group in all the measures assessed in the post-asssessment and at follow-up.

Similarly, there was a significant decrease in BIDs, including body distortion and body dissatisfaction levels, particularly among the patients in the experimental group. These findings are particularly important, since patients with AN usually present persistent cognitive and emotional disturbances in the way they perceive their body weight or shape [16]. Moreover, previous research suggests that patients with long-term BIDs have a higher risk of future relapse and more negative outcomes after treatment [78].

Our results also support previous studies that used in-vivo body exposure procedures (i.e., MET) to reduce body dissatisfaction among patients with EDs [79] and patients with AN [36]. However, the previous studies exposed individuals to their real body over a prolonged period of time to reduce negative body-related responses (such as a negative mood or body dissatisfaction) through habituation [36,37,80], while the current study went one step further and first exposed patients to their real-size virtual body, and then applied a gradual increase in their BMI over successive sessions until they owned a virtual avatar with a healthy BMI. Thus, VR technology and embodiment-based procedures allow us to go further and help patients with AN reduce their negative body-related responses, such as body dissatisfaction, not only when they are exposed to their real body (as has been previously done in MET), but also when they are exposed to a virtual representation of their own body with a certain amount of weight gain.

Patients in the experimental group also showed a significant reduction in body distortion levels at follow-up. These findings are important, since body distortion is one of the key components targeted in the treatment of AN and an improvement is critical for a better long-term outcome among adolescents with AN [81]. However, there are only a few treatment approaches directly targeting body distortion or assessing changes before and after usual inpatient treatment in AN [82]. One key aspect of using a VR embodiment-based procedure is that it allows individuals to realistically experience a virtual body as their own body, eliciting the same sensorial responses or activating the same implicit or explicit multisensory representations of their own body [83,84]. Moreover, the modification of the size or shape of the virtual body can be used to improve the disturbed representation of the body, as has been reported in patients with AN [46,52] and patients with other mental health issues [84]. The current study is the first to elicit changes in body distortion after several repeated treatment sessions, in contrast to previous studies that reduced body distortion levels in patients with AN after one single session of exposure to a standard healthy body [46,52]. Furthermore, the weight gain of their avatar was spread across the virtual body that initially represented the patient’s silhouette and BMI. Thus, it allowed more accurate and realistic weight increases over successive sessions. This could have been a key factor in helping patients internalize the changes in their own real bodies after the intervention. Indeed, our results on the FBI levels support this. FBI levels in the experimental group were notably higher in the post-assessment than in the pre-assessment, while no changes were observed in the control group. A previous study demonstrated that higher body distortion levels significantly predicted lower FBI levels among patients with AN [69]. Thus, this relationship might be bidirectional, meaning that regarding the virtual body progressively more as their own body might have helped patients with AN correct their biased perception of their own body size, thereby reducing body distortion levels.

After the intervention, patients in the experimental group showed lower AB towards W-AOIs than those in the control group, who showed higher AB towards W-AOIs. These tendencies, even if not significant, suggested that body-related AB in patients with AN might be modified by a VR-based body exposure intervention. A key aspect in understanding these results might rely on the procedure conducted in the experimental group, in which patients attended at different parts of the virtual body (from the head to the shoes), while they were asked to orally express what they thought and felt about those body areas. Thus, since patients had to look at those parts for a certain amount of time in every session, this could have reduced their AB towards weight-related body areas, leading the patients to spread their attention more equally among all the body parts. Indeed, similar procedures have been conducted in previous studies with women presenting high body dissatisfaction, in which a short-term AB towards (un)attractive body areas was induced to reduce body dissatisfaction levels among healthy women [85,86,87]. However, to the best of our knowledge, the current study is the first to specifically assess body-related AB among AN patients before and after a body exposure-based intervention. These results, nevertheless, should be carefully considered, since eye-gaze behavior was not properly controlled during the sessions (e.g., by using ET devices) and the sample size was small (only 23 patients were assessed using ET devices before and after the intervetion). Modifing body-related AB is an important target in ED treatments [88]. Several studies suggest a bidirectional relationship between body-related AB and other body-related concerns, especially body dissatisfaction in patients with EDs [21,58] and also among adolescents with AN [23].

Finally, preliminary evidence suggested that age only emerged as a significant predictor of higher body dissatisfaction levels post-intervention in the experimental group. This indicated that the VR exposure intervention was more effective in reducing body dissatisfaction among young patients compared to adult patients. Indeed, adult patients reported lower FBI levels than younger patients, specifically in the post-assessment, which could be a key aspect to target in order to reduce other symptoms such as body dissatisfaction and improve overall treatment efficacy. In fact, a previous study that used a digital variant of the RHI found that age was directly associated with a reduced perceptual illusion over the fake hand [89]. Thus, similar results could be expected when the ownership illusion is elicited over a whole virtual body. Furthermore, our results are in line with those of another study that found that it was more difficult to elicit changes in body representations among adults compared to young healthy participants [90]. The current study, thus, expands previous findings in a clinical sample of patients with AN, suggesting that future studies should offer tailored interventions separately to adolescents and adults with AN.

The current study had some limitations that should be addressed in future studies. Regarding methodological limitations, the sample size was small and, thus, the current findings should be considered carefully. Although a prior power analysis suggested the need for a higher sample size (i.e., 54 patients), it was not possible to achieve this figure. Furthermore, due to the small sample size, data from both adults and adolescents with AN were considered together in the statistical analyses. However, preliminary evidence indicated that age was significantly associated with a worse clinical outcome in the experimental group. Consequently, future research should assess the efficacy of the current intervention separately for adults and adolescents with AN.

Statistical analyses were conducted without controlling for some key variables, such as the sub-type of AN, illness duration, other mental health comorbidities, and pharmacological treatments. In addition, the current study only had a three-month follow-up, which might not have been long enough to observe the complete development of ED symptoms over a long period of time or to assess future relapses among patients. Future studies could attempt to replicate or extend the current findings with a longer follow-up assessment (for instance, one-year follow-up). In addition, all our patients attended day patient programs, and so it may not be possible to generalize our results to more severe cases of AN who usually attend intensive 24 h-hospitalization programs. Finally, although the drop-out rates of the current study are low, particularly among patients in the experimental group (where only two patients abandoned), it would have been useful to complement this information with a more accurate assessment of satisfaction with treatment among patients.

The other limitations were associated with the AN-VR-BE software. These limitations should be considered and improved in the future updates of the software. Despite realistically reproducing a virtual body with the same silhouette and BMI as that of the patient’s body, other important features of the general appereance (e.g., the general outfit, the tone of the skin, the hairstyle, etc.) could not be simulated. Furthermore, the adult patients usually reported that the virtual body seemed “too young” for them, which could also explain the lower FBI levels found among them compared to the adolescent patients. A possible way to improve the realism of the avatar is by using 3D scans and biometric avatars, which allow the personalization of virtual bodies with all the features of the individual [70]. Thus, future studies can reproduce current findings using highly realistic virtual bodies and different virtual environments to generalize the current findings to other highly relevant everyday life situations (e.g., dressing rooms, swiming pools, beaches, etc.).

Although evidence-based treatments for AN have significantly improved in quality and amount in the last decade, further research is still needed to improve the interventions for AN, including CBT, which often achieves modest results [4]. The randomized clinical trial presented in this study showed that a VR exposure therapy can enhance the effectiveness of the usual day-patient treatment for AN and help patients with AN confront their core FGW. Specifically, our results showed that patients in the experimental group showed significantly reduced FGW and BID levels than those in the control group after the intervention and at follow-up. Furthermore, promising non-significant trends were observed for other ED measures, such as the BMI, body anxiety, drive for thinness, body appreciation and body-related AB.

The use of VR technology might improve research and clinical practice in AN by providing new tools to help patients confront their core fears (i.e., food- or weight-related cues) and improve their emotional, cognitive, and behavioral responses to their body image as well as other important ED symptoms. Future research might test the implications of the current findings in more severe cases of AN. As stated above, the use of in-vivo mirror and body exposure techniques in severely emaciated AN patients is controversial, as it may elicit habituation toward extremely low weights. Future VR body exposure interventions should try to provide innovative solutions to this issue. Rather than helping patients with AN to habituate toward their own body, this technology might be used to help them gradually cope with their core fears, such as weight gain. However, some important challenges remain, for example, improving adherence at this early stage of the treatment, and adapting a specific (VR) body exposure procedure in which patients with severe AN do not have to stand for a long period of time. Furthermore, combining VR and ET technologies might lead to new possibilities in the field of body-related AB in EDs. For example, patients could be immersed in VR environments that simulate everyday life situations that are emotionally salient for them (i.e., expose their body in a gym, a beach, a pool, etc.), with their gaze patterns continuously measured using ET devices. This could provide an objective measure of how patients with EDs or AN spontaneously interact with the clinical stimuli (e.g., self vs. other avatars with different body sizes or faces expressing positive vs. negative emotions) within the VR environment. Additionally, clinical interventions could use our findings to design specific interventions that aim to retrain dysfunctional body-related AB among patients with AN or other EDs.

To conclude, the current study presents an innovative and pioneering VR body exposure procedure that could have promising future applications in the field of EDs and BIDs.

## Figures and Tables

**Table 1:** Descriptive results, including the demographic and clinical characteristics of both groups.

Variable	Experimental Group (*n =* 16)	Control Group (*n =* 19)	*X* ^2^ *(df)*	*t-Test (df)*	*p Value*
Age. Mean years (SD)	18.25 (1.30)	19.21 (1.78)	N/A	0.419	0.68
**Group age**, *n* (%)			0.551 (1)	N/A	0.5
Adolescents	9 (56.25)	12 (63.16)			
Adults	7 (43.75)	7 (36.84)			
BMI ^a^, mean kg/m^2^ (SD)	17.30 (1.06)	17.54 (1.27)	N/A	0.513	0.44
*Sex, n (%)*			*0.033 (1)*	*N/A*	*>0.99*
Women	14 (87.5)	17 (89.47)			
Men	2 (12.5)	2 (10.52)			
**Main diagnosis**, *n* (%)			0.285 (2)	N/A	0.7
AN-R type ^b^	13 (81.3)	14 (73.7)			
AN-P type ^c^	3 (18.8)	5 (26.3)			
**Comorbid diagnosis**, *n*			N/A	N/A	N/A
GAD ^d^	1	0			
MDD ^e^	1	1			
PDD ^f^	1	0			
BPD ^g^	2	2			
PTSD ^h^	1	0			
Anxiety NOS ^i^	1	1			
Depression NOS	0	2			
**Number of comorbid diagnosis**, *n* (%)			0.810 (2)	N/A	0.84
0	10 (68.6)	14 (77.4)			
1	5 (31.3)	4 (21.1)			
2	1 (6.3)	1 (5.3)			
**Medication**			2.789 (4)	N/A	0.69
None	7	12			
Antidepressant	2	3			
Anxiolytic	2	1			
Antidepressant and Anxiolytic	2	2			
Antipsychotic + Antidepressant/Anxiolytic	3	1			

Note: N/A: not applicable. ^a^ BMI: body mass index, ^b^ AN-R: anorexia nervosa restrictive subtype, ^c^ AN-P: anorexia nervosa purgative subtype, ^d^ GAD: generalized anxiety disorder, ^e^ MDD: major depressive disorder, ^f^ PDD: persistent depressive disorder, ^g^ BPD: borderline personality disorder, ^h^ PTSD: posttraumatic stress disorder, ^i^ NOS: not otherwise specified.

## Data Availability

The data presented in this study are available on request from the corresponding author. The data are not publicly available due to patients’ privacy restrictions.
